# Editorial to the Special Issue ‘Nutritional Intervention in Digestive Diseases in the Era of Nutraceuticals, Nutrigenomics and Microbiomics’

**DOI:** 10.3390/nu15132964

**Published:** 2023-06-29

**Authors:** Hassan Ashktorab, Hassan Brim

**Affiliations:** Department of Medicine, Gastroenterology Division, Department of Pathology and Cancer Center, Howard University College of Medicine, Washington, DC 20059, USA; hbrim@howard.edu

Environmental exposures, particularly diet, play an important role in the prevention or exacerbation of illnesses, including gastrointestinal (GI) diseases. This statement goes along with the knowledge that “we are what we eat” and that food and nutrients are significant contributors or factors in the health of the GI system. Ingested food supplies the body with multiple necessary nutrients, metabolites and compounds that have systemic effects. As such, nutritional interventions that take into consideration the health status of the patient, type and stage of the diseases in general and the gastrointestinal ones in particular as well as the patient’s genetics and genomics are needed in order to have effective and targeted nutritional interventions to reverse and/or mitigate diseases’ conditions. In the present issue, many such cases have been discussed.

Stefan Lucian Popa et al.’s paper [1] addressed the issue of functional dyspepsia, a common and prevalent disorder of the brain–gut interaction that impacts patients’ quality of life and has limited response to traditional pharmacological agents. Recently, many groups have studied the importance of various psychological therapies and nutritional recommendations for these patients. Food intolerances were found to be correlated with this disorder in many patients, leading to patients’ adoption of exclusion diet strategies to eliminate specific compounds from their diet. Popa et al.’s systematic review analyzed the impact and efficiency of certain exclusion diets undertaken by patients, more precisely, the gluten-free diet and the low-FODMAP diet.

The low-FODMAP (fermentable oligosaccharides, disaccharides, monosaccharides and polyols) diet was also studied by Peng et al. [2] to assess its impact on functional gastrointestinal symptoms (FGS) and other diseases’ features in inflammatory bowel disease (IBD) patients with functional GI symptoms. Peng et al. used and screened six patient databases to collect relevant data. The analysis involved a total of 446 participants (351 patients with low-FODMAP diet and 95 controls). Peng et al.’s analysis showed that the low-FODMAP diet led to an improvement in FGSs and QoL but not in stool consistency or mucosal inflammation in IBD patients. This analysis showed that diet intervention might be added as a treatment strategy in IBD patients with FGS.

Stephen J. Allen et al. [3] analyzed dietary therapy as a means to improve nutrition and gut health in pediatric Crohn’s disease patients. They assessed the effect of bovine colostrum (BC), which is known to have anti-inflammatory, anti-infective, growth and intestinal repair factors of potential benefit in Crohn’s disease (CD). Daily BC for up to 3 months was given to children and young people with CD in remission or of mild/moderate severity. In interviews with patients included in this clinical trial, opinions were divided as to preference for BC over the placebo milk, and some preferred BC over other nutritional supplements. Symptoms, clinical and laboratory variables and quality of life were similar in the two arms. Due to its anti-inflammatory properties, BC may be an acceptable nutritional supplement for daily, longer-term use in young patients with CD.

Advanced incurable disease stages generally lead to surgeries that are associated with diseased section removal, such as colectomy in the context of colon cancer. Such interventions in the GI system need to be accompanied by dietary and nutritional interventions to account for the resected GI sections. In this issue, Antonio Fernández-Gálvez et al. [4] addressed this issue in the context of ileostomy. They evaluated a diet intervention in order to determine its capacity to improve the eating pattern of patients with an ileostomy, facilitating the implementation of new eating-related behaviors and reducing doubt and dissatisfaction and other complications. The nutritional intervention consisted of a duly modified Mediterranean-diet-based set of menus that was reinforced by specific counseling at the reintroduction of oral diet, hospital discharge and first follow-up appointment. Most patients had a favorable experience regarding weight recovery and a significant reduction in all-cause readmissions and readmission with dehydration. The intervention helped the effective self-management of eating patterns by patients.

Anti-inflammatory diets’ impact in ulcerative colitis was investigated by Ammar Hassanzadeh Keshteli et al. [5] Indeed, a relationship between UC and diet has been shown in several studies. The menu plans in the anti-inflammatory diet were rich in dietary fiber, probiotics, antioxidants and omega-3 fatty acids and had a low intake of red meat, processed meat and added sugar. Fecal calprotectin and microbiota testing, along with metabolomic analysis of urine, serum and stool samples, were performed. Their study showed that dietary modifications involving the increased intake of anti-inflammatory foods combined with a decreased intake of pro-inflammatory foods were associated with metabolic and microbial changes in UC patients in clinical remission and were effective in preventing subclinical inflammation.

Curcumin is a traditional spice of interest that is known to have many beneficial properties. In this issue, Nazli Gullii et al. [6] analyzed its anti-tumor effects in the context of MACC1 gene-driven tumors’ metastasis. Metastasis is the main reason for the high mortality rate of colorectal cancer (CRC) patients. They aimed to assess MACC1 inhibition using natural products due to their low side effects. Curcumin reduced the MACC1 expression, restricted the MACC1-induced proliferation and was able to reduce the MACC1-induced cell motility as one of the crucial steps for the distant dissemination of the tumor. A MACC1-dependent effect of curcumin on clonogenicity and wound healing was also demonstrated. This was the first identification of the effect of curcumin on the restriction of cancer motility, proliferation and colony-forming ability via MACC1 as a target.

Specific food additives with anti-inflammatory properties can also have similar effects to anti-inflammatory food regimens. Indeed, we have previously shown in three animal models that saffron has major effects on the gut microbiome in a rat model and major anti-inflammatory, microbiome, metabolome and immune-modulating properties in DSS and TNBS mouse models of colitis [7]. Saffron intake before and after DSS administration showed protective and healing effects, respectively. These findings reflect that saffron can be used to prevent inflammation and to revert ongoing established inflammation. These findings were further validated in a clinical trial where UC patients taking saffron had lower inflammation, as reflected by lower stool calprotectin levels. [8,9]. These findings prompted us to dissect the potential mechanisms of action of saffron’s active components. In this issue, Mudasir et al. [10] reported on multiple components, such as carotenoids as bioactive dietary phytochemicals, which can directly or indirectly regulate epigenetic factors and alter gene expression profiles. Previous reports have shown the interaction between active saffron compounds with linker histone H1. Other reports have shown that high concentrations of saffron bind to the minor groove of calf thymus DNA, resulting in specific structural changes from the B- to the C-form of DNA. Moreover, the interaction of crocin G-quadruplex was reported. A recent in silico study has shown that residues of SIRT1 interact with saffron’s bio-active compounds and might enhance SIRT1 activation. Other reports have shown that the treatment of saffron’s bio-active compounds increases H2AX and decreases HDAC1 and phosphorylated histone H3 (p-H3). Further integrative studies will shed more light on the connections between saffron’s epigenetic effects and its other effects mentioned above (microbiome, metabolome and immunological).

The studies and review papers in this issue reflect the multitude of nutritional interventions that range from exclusion diets to specific diet regimens to food additives and supplements. All of these interventions need to be tailored to specific goals and will vary in terms of whether they are administered to healthy subjects for preventive purposes or to patients with different diseases in general and different GI diseases in particular. Next-generation nutritional interventions need to be multifaceted and must include the use of well-established nutraceuticals with known properties, nutrigenomics, which should account for populations and individual genomic profiles, and microbiome profiling, as the gut microbiome is a major actor in the interface between ingested food and host genetics.
